# Evaluation of selected tropical marine microalgal cultures for use in biophotovoltaic platforms

**DOI:** 10.1007/s00253-023-12951-0

**Published:** 2024-01-09

**Authors:** Zoe Hui-Yee Tay, Fong-Lee Ng, Cheng-Han Thong, Choon-Weng Lee, G. Gnana kumar, Abdullah G. Al-Sehemi, Siew-Moi Phang

**Affiliations:** 1 Institute of Biological Sciences, Faculty of Science, Universiti Malaya, Kuala Lumpur, Malaysia; 2 Institute of Ocean and Earth Sciences (IOES), Universiti Malaya, Kuala Lumpur, Malaysia; 3 School of Biosciences, Taylor’s University, Lakeside Campus, 47500 Subang Jaya, Selangor Darul Ehsan Malaysia; 4 Institute for Advanced Studies (IAS), Universiti Malaya, Kuala Lumpur, Malaysia; 5 Department of Physical Chemistry, School of Chemistry, Madurai Kamaraj University, Madurai, 625021 Tamil Nadu India; 6 Faculty of Engineering Technology & Built Environment, UCSI University, 56000 Kuala Lumpur, Malaysia; 7 Research Center for Advanced Materials Science (RCAMS), King Khalid University, 61413 Abha, Saudi Arabia; 8 Faculty of Applied Sciences, UCSI University, Kuala Lumpur, Malaysia

**Keywords:** Renewable energy, Bioelectricity, Algal biotechnology, Marine microalgae, Biophotovoltaic platform

## Abstract

**Abstract:**

In this study, the bioelectrical power generation potential of four tropical marine microalgal strains native to Malaysia was investigated using BPV platforms. *Chlorella* UMACC 258 produced the highest power density (0.108 mW m^−2^), followed by *Halamphora subtropica* UMACC 370 (0.090 mW m^−2^), *Synechococcus* UMACC 371 (0.065 mW m^−2^) and *Parachlorella* UMACC 245 (0.017 mW m^−2^). The chlorophyll-a (chl-a) content was examined to have a linear positive relationship with the power density (*p* < 0.05). The photosynthetic performance of strains was studied using the pulse-amplitude modulation (PAM) fluorometer; parameters measured include the following: maximum quantum efficiency (*F*_v_/*F*_m_), alpha (*α*), maximum relative electron transport rate (rETR_max_), photo-adaptive index (*E*_k_) and non-photochemical quenching (NPQ). The *F*_v_/*F*_m_ values of all strains, except *Synechococcus* UMACC 371, ranged between 0.37 and 0.50 during exponential and stationary growth phases, suggesting their general health during those periods. The low *F*_v_/*F*_m_ value of *Synechococcus* UMACC 371 was possibly caused by the presence of background fluorescence from phycobilisomes or phycobiliproteins. Electrochemical studies via cyclic voltammetry (CV) suggest the presence of electrochemically active proteins on the cellular surface of strains on the carbon anode of the BPV platform, while morphological studies via field emission scanning electron microscope (FESEM) imaging verify the biocompatibility of the biofilms on the carbon anode.

**Key points:**

*• Maximum power output of 0.108 mW m* ^*−2*^ *is recorded by Chlorella UMACC 258*

*• There is a positive correlation between chl-a content and power output*

*• Proven biocompatibility between biofilms and carbon anode sans exogenous mediators*

**Supplementary Information:**

The online version contains supplementary material available at 10.1007/s00253-023-12951-0.

## Introduction

Continuous consumption of fossil fuels to generate electricity has led to their steady depletion, rise in greenhouse gas (GHG) emissions and global warming. While numerous efforts have been made to derive energy from renewable sources such as tides, winds and geothermal heat (Kammen 2006; Gielen et al. 2019), the process of converting such forms of energy into electrical energy still involves the generation of carbon footprint, particularly carbon dioxide (CO_2_) (Adams and Nsiah 2019). The global CO_2_ emissions reached an all-time high of 33 gigatonnes (Gt) in 2019 and only experienced a decline to 31.5 Gt due to the COVID-19 pandemic in 2020 (IEA 2021).

The study of the exploitation of photoautotrophic microorganisms in photosynthetic microbial fuel cells (PMFCs) has become increasingly popular in recent decades due to their ability to fixate atmospheric CO_2_ (Venkata Mohan et al. 2014; Lee et al. 2015; Kusmayadi et al. 2020), so no net CO_2_ is produced in this system. In addition, microalgae are able to sequestrate CO_2_ more efficiently than terrestrial plants (Jaswal et al. 2020) and have other favourable characteristics such as fast growth rates, ability to adapt in hostile environments and produce diverse bioproducts (Demirbas and Demirbas 2010). In addition, their cultivation does not require the use of arable land unlike terrestrial plants (Saifullah et al. 2014).

To generate bioelectricity by utilising algae, a biophotovoltaic (BPV) platform is used. It is a subcategory of PMFC which utilises the photosynthetic process of microalgae to turn solar energy into electrical energy (Mccormick et al. 2011). When oxygenic microalgae absorb light, they carry out water-splitting reactions to release oxygen (O_2_), protons (H^+^) and electrons. The electrons are then harvested through an anode to create bioelectricity (Thong et al. 2023).

Exogenous mediators were used in earlier algal BPV studies to enable electron transfer (Tschörtner et al. 2019) but were later realised to be toxic to microorganisms and the environment. They were also unsuitable for long-term operation of the BPV platform (Bombelli et al. 2011). The discovery of biofilm development on the electrode proved to be a simpler and more appropriate alternative to the use of exogenous mediators (Mccormick et al. 2011). According to Ng et al. (2014a, b), biofilm development could provide “direct contact between cell and electrode and reduced internal potential losses”, and in turn, lead to greater power output.

As for anode material, indium tin oxide (ITO) coated glass is commonly used to facilitate algal biofilm growth (Mccormick et al. 2011). Reduced graphene oxide (rGO) is an effective alternative material reported to provide a 119% increment in efficiency when compared to ITO (Ng et al. 2014a). Another anode material reported was ruthenium(IV) oxide (RuO_2_)/tungsten(VI) oxide (WO_3_) composite nanofibers. RuO_2_/WO_3_ could improve the structural stability of the platform, whereas the nanofibers could aid with the reduction in internal resistance of the platform (Karthikeyan et al. 2019; Tee et al. 2023). Furthermore, Ng et al. (2017) found that the immobilisation of algal cultures could generate even greater power output than suspension cultures.

Nevertheless, to the best of our knowledge, tropical marine microalgae native to Malaysia have not been investigated for their use in BPV platforms. Therefore, the aim of this study is to evaluate the bioelectricity generation potential of various tropical marine microalgal strains through the use of BPV platforms.

To achieve the aim, microalgae suspension cultures were grown on carbon paper electrodes within the BPV platforms. The microalgal strains tested were chlorophytes *Parachlorella* UMACC 245 and *Chlorella* UMACC 258, diatom *Halamphora subtropica* UMACC 370 and cyanobacterium *Synechococcus* UMACC 371. Upon completing the BPV platform set-up, electrical measurements were taken. The experimental irradiance level used was 90 µmol photons m^−2^ s^−1^. Biocompatibility of carbon anodes in the BPV platforms was then analysed using the field emission scanning electron microscope (FESEM). Microalgae growth was assessed based on chlorophyll-a (chl-a) analysis, photosynthetic performance using pulse-amplitude modulation (PAM) fluorometry and the redox nature, current production and electrokinetics using cyclic voltammetry (CV) (Busalmen and de Sánchez 2005; Ng et al. 2017).

It is hypothesised that these algal strains would possess some photosynthetic ability capable of the harvest of light energy and their conversion into electrical energy. Data obtained from this study could enhance our knowledge on the photosynthetic performance, bioelectricity generation potential and redox activity of the selected marine algal strains and widen our choices of algal strains usable in BPV platforms.

## Material and methods

### Microalgal cultures

The strains selected for this study were *Parachlorella* UMACC 245, *Chlorella* UMACC 258, *Halamphora subtropica* UMACC 370 and *Synechococcus* UMACC 371 from the University of Malaya Algae Culture Collection (UMACC). They were selected based on characterisation studies including the parameters of photosynthetic performance and growth rate (Lim et al. 2018).

Exponential phase cultures standardised at optical density (OD) of 1.0 at 620 nm (OD_620nm_ = 1.0) were inoculated at 20% inoculum size. The algal cultures were grown in Prov medium in 250 mL conical flasks (total volume: 100 mL). Cultures were placed on an incubator shaker (YIH DER, Model: TS-520D) at 25 °C, 120 rpm with irradiance of 30 µmol photons m^−2^ s^−1^ on a 12:12 light–dark cycle (Thong et al. 2023).

### BPV platform set-up and electrical measurement

**Fig. 1:**
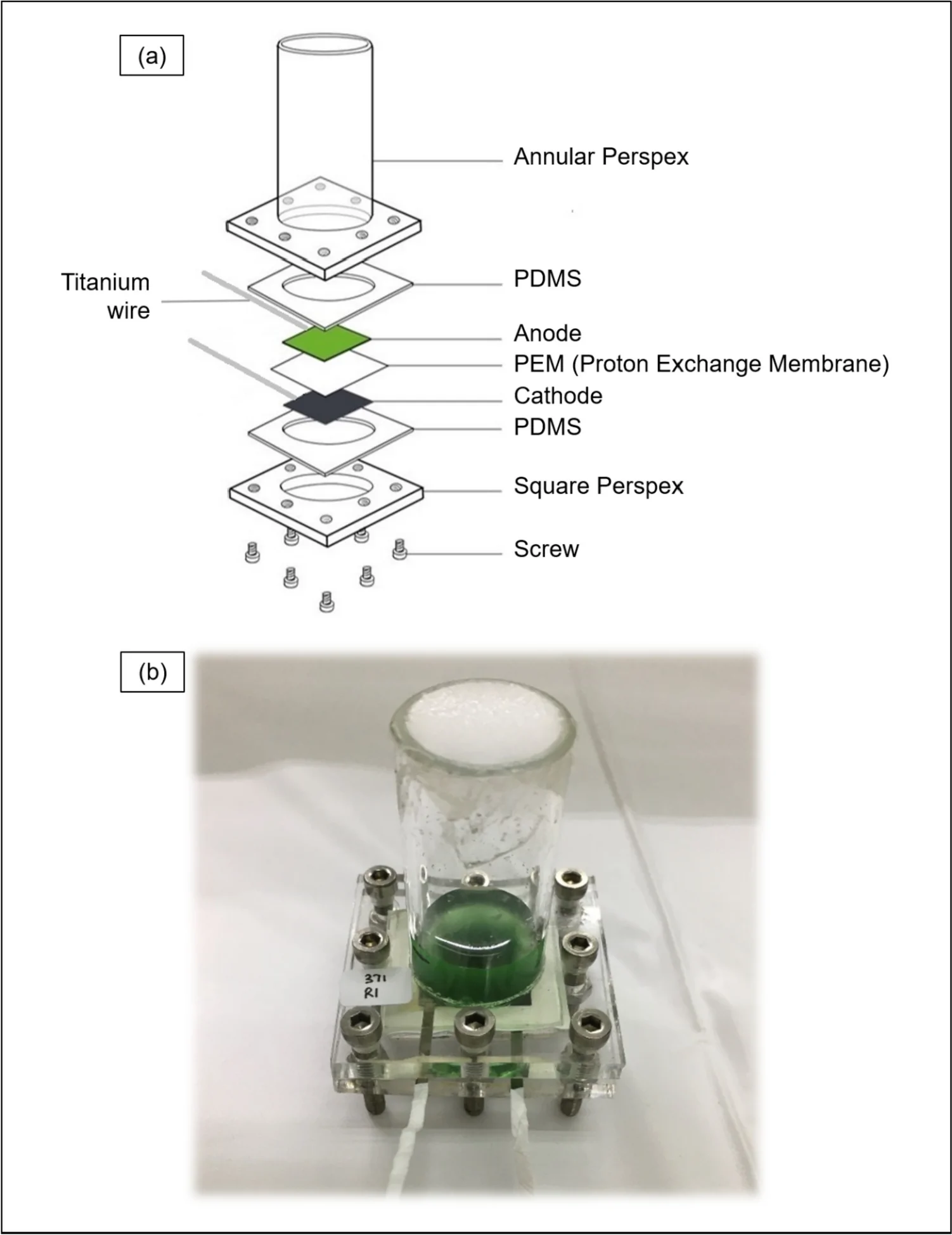
**a** Exploded view of a BPV platform showing the individual parts that make up a BPV; **b** example of a BPV platform containing algal culture with its annular inlet sealed with sterile mesh dressing

Both cathode and anode used are made of carbon paper (Alfa Aesar, USA). Algal suspension cultures were loaded onto the anode through an open inlet in the centre of the BPV platform. The inlet was then sealed with a sterile mesh dressing (Smith & Nephew, UK). A nitrocellulose membrane was placed in between the cathode and anode to act as an ion bridge, after which crocodile clips and titanium wires were used to connect electrodes to the external circuit (Fig. 1).

Each microalgal strain was exposed to an irradiance of 90 µmol photons m^−2^ s^−1^ using a photometer (LI-COR Biosciences, Model: LI-250A) for the duration of the experiments. BPV temperature was maintained at 25 °C. Power outputs were measured using a multimeter (Agilent U1251B) in the measurement unit of millivolt (mV). Various resistance values (10 MΩ, 6.8 MΩ, 5.6 MΩ, 2 MΩ, 560 kΩ, 240 kΩ, 62 kΩ, 22 kΩ, 9.1 kΩ) were applied to the external circuit (Ng et al. 2018; Thong et al. 2019). By applying Ohm’s law, polarisation curves were obtained so as to calculate the maximum current density and maximum power density (Logan et al. 2006). All experiments were conducted in quintuplicates. Statistical analyses were conducted using the SPSS programme. A Control (with only Prov medium) and no microalga inoculum were also set up in quintuplicate to measure the power output of the medium only.

### Pigment content determination

#### Chlorophyll-a (Chl-a)

On days 0, 4, 8 and 12 of the experiment, microalgae were removed from the anode, and their chl-a content was determined via the spectrophotometric method (Strickland and Parsons 1968) to estimate algal biomass and indicate the growth rate of algal cells (Ng et al. 2017).

Algal cells were removed from the carbon paper anodes and filtered onto glass-fibre filter paper (Whatman GF/C, 0.45 µm). The papers were mashed using a tissue grinder (Kimble, USA) and soaked with 10 mL of analytical grade 100% acetone. Samples were kept in a freezer for 24 h at 4 °C before being centrifuged at 3000 rpm, 4 °C for 10 min. Absorption of supernatant was measured at 630 nm (OD_630nm_), 645 nm (OD_645nm_) and 665 nm (OD_665nm_). The chl-a concentration was calculated using the equation:

1 $$Chl-a\;\left(mg\;m^{-3}\right)=\frac{C_a\times V_a}{V_c}$$

where

*C*_a_ =: $$11.6\times{OD}_{665nm}-1.31\times{OD}_{645nm}-10.14\times{OD}_{630nm}$$  
*V*_a_ =: volume of acetone used for extraction (mL)
*V*_c_ =: volume of culture (L)

$$Chl-a\;\left(mg\;L^{-1}\right)=\frac{Chl-a\;\left(mg\;m^{-3}\right)}{1000}$$

#### Total carotenoid

Carotenoid is an accessory pigment which also aids in light-harvesting activities in addition to providing protection against photooxidative damage (Henríquez et al. 2016). Total carotenoid content was determined using the spectrophotometric method at a wavelength of 452 nm. The equation used for carotenoid content calculation is as follows:

2 $$Total\;carotenoid\;(mg\;m^{-1})=\frac{{OD}_{452nm}\times3.86\times V_e}{V_c}$$

where *V*_e_ = volume of extract (acetone in mL); *V*_c_ = volume of culture (L).

#### Phycobiliproteins (PBP)

Similar to chl*-*a and carotenoid extraction, PBP content was determined spectrophotometrically at wavelengths of 562 nm (OD_562nm_), 615 nm (OD_615nm_) and 652 nm (OD_652nm_) for the blue-green microalgal strain, *Synechococcus* UMACC 371. PBPs are accessory pigments found in cyanobacteria and red algae. They are subcategorised into phycocyanin (blue pigment), allophycocyanin (light-blue pigment) and phycoerythrin (red pigment) (Christaki et al. 2015). The extract used was 0.01 M NaHPO4 solution containing 0.15 M NaCl at pH 10. The equations used to calculate the respective type of PBP are as follows (Bennett and Bogorad 1973):

3 $$\mathrm{Phycocyanin}\;\lbrack PC\rbrack\;(mg\;L^{-1})=\frac{{OD}_{615nm}-0.474\times{OD}_{652nm}}{5.34}$$

4 $$\mathrm{Allophycocyanin}\;\lbrack APC\rbrack\;(mg\;L^{-1})=\frac{{OD}_{652nm}-0.208\times{OD}_{615nm}}{5.19}$$

5 $$\mathrm{Phycoerythrin}\;\lbrack PE\rbrack\;(mg\;L^{-1})=\frac{{OD}_{562nm}-2.41\;\left[PC\right]-0.849\;\lbrack APC\rbrack}{9.62}$$

The concentration of PC, APC or PE content in a given volume of culture was determined using the following equation:

6 $$PC\;or\;APC\;or\;PE\;(mg{\;m\;L}^{-1}in\;the\;culture\;cells)=\frac{C\times V_e}{V_c}$$

where

*C =*: concentration of PBP (PC or APC or PE)
*V*_e_ =: volume of extract (mL)
*V*_c_ =: volume of culture (mL)

### Pulse amplitude modulation (PAM) fluorometry measurement

Photosynthetic parameters were measured fluorometrically using a Diving-PAM (Walz, Germany) (McMinn et al. 2010) to assess the photosynthetic efficiency of the light-harvesting pigments in photoautotrophic microorganisms (Ciniciato et al. 2016). The photosynthetic parameters to be investigated include maximum quantum efficiency (*F*_v_/*F*_m_), maximum relative electron transport rate (rETR_max_), photo-adaptive index (*E*_k_) and non-photochemical quenching (NPQ).

Rapid light curves (RLCs) were derived under software control (Wincontrol, Walz). First, algal cultures were dark-adapted for 15 min. *F*_v_/*F*_m_ was deduced using the formula as follows:

7 $${F}_{v}/ {F}_{m}=\frac{{(F}_{m}-{F}_{0})}{{F}_{m}}$$

where

*F*_m_ =: maximum fluorescence value
*F*_0_ =: minimum fluorescence value

Maximum photosynthetic efficiency was established from the initial slope (*α*) of RLC. The relative electron transport rate (rETR) was found by multiplying the irradiance by the quantum yield measured at the end of each light interval. *E*_k_ was calculated using the formula *E*_k_ = rETR_max_/*α* where rETR_max_ is the maximum relative electron transport rate. Statistical analyses were carried out using the SPSS programmme.

### Cyclic voltammetry (CV) measurement

CV measurement was carried out using a three-electrode system with the anode of the BPV platform as the working electrode, cathode as the counter electrode and the Ag/AgCl electrode in 3 M KCl solution as the reference electrode. A range of potentials between 0 and + 1 V was applied to the anode using a potentiostat (Metrohm Autolab PGSTAT204, Netherlands). All electrochemical measurements on the algal BPV platforms were performed with the same potentiostat. All potentials were reported against the reference. CV was carried out within a pre-determined potential range and scan rate to determine the oxidation–reduction potential of the biofilm (Busalmen and de Sánchez 2005). All electrochemistry procedures were carried out at a constant temperature of 25 °C.

### Morphological studies

Surface morphology of the microalgal cells on the carbon anodes was studied by using FESEM. Algal biofilms were grown on the carbon anode, after which critical point drying (CPD) was carried out according to established protocol (Anderson 1951). FESEM imaging was done on the samples to examine the growth of the microalgal biofilms on the carbon paper anodes and determine the biocompatibility between the two.

## Results

### Power output generated in BPV platforms

#### Polarisation curves during the exponential phase of algal growth

**Fig. 2:**
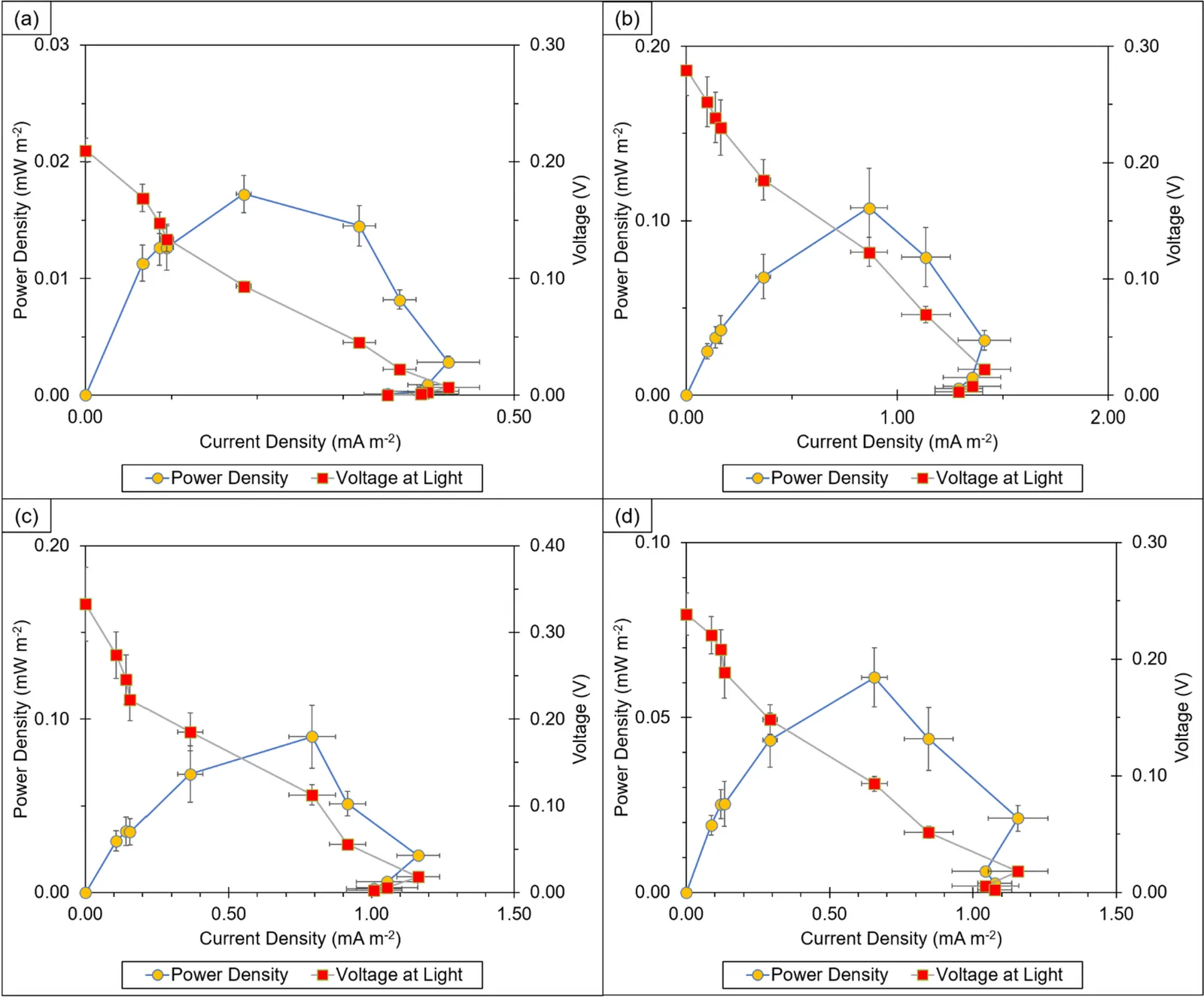
Polarisation curves of **a** *Parachlorella* UMACC 245, **b** *Chlorella* UMACC 258, **c** *Halamphora subtropica* UMACC 370 and **d** *Synechococcus* UMACC 371 on Day 4; data as means ± S.D (*n* = 5)

Figure 2 shows the polarisation curves plotted to determine the maximum current densities and power densities of all four tropical marine strains on Day 4, when algal cell numbers had increased exponentially.

#### Comparison between maximum power densities of strains

**Fig. 3:**
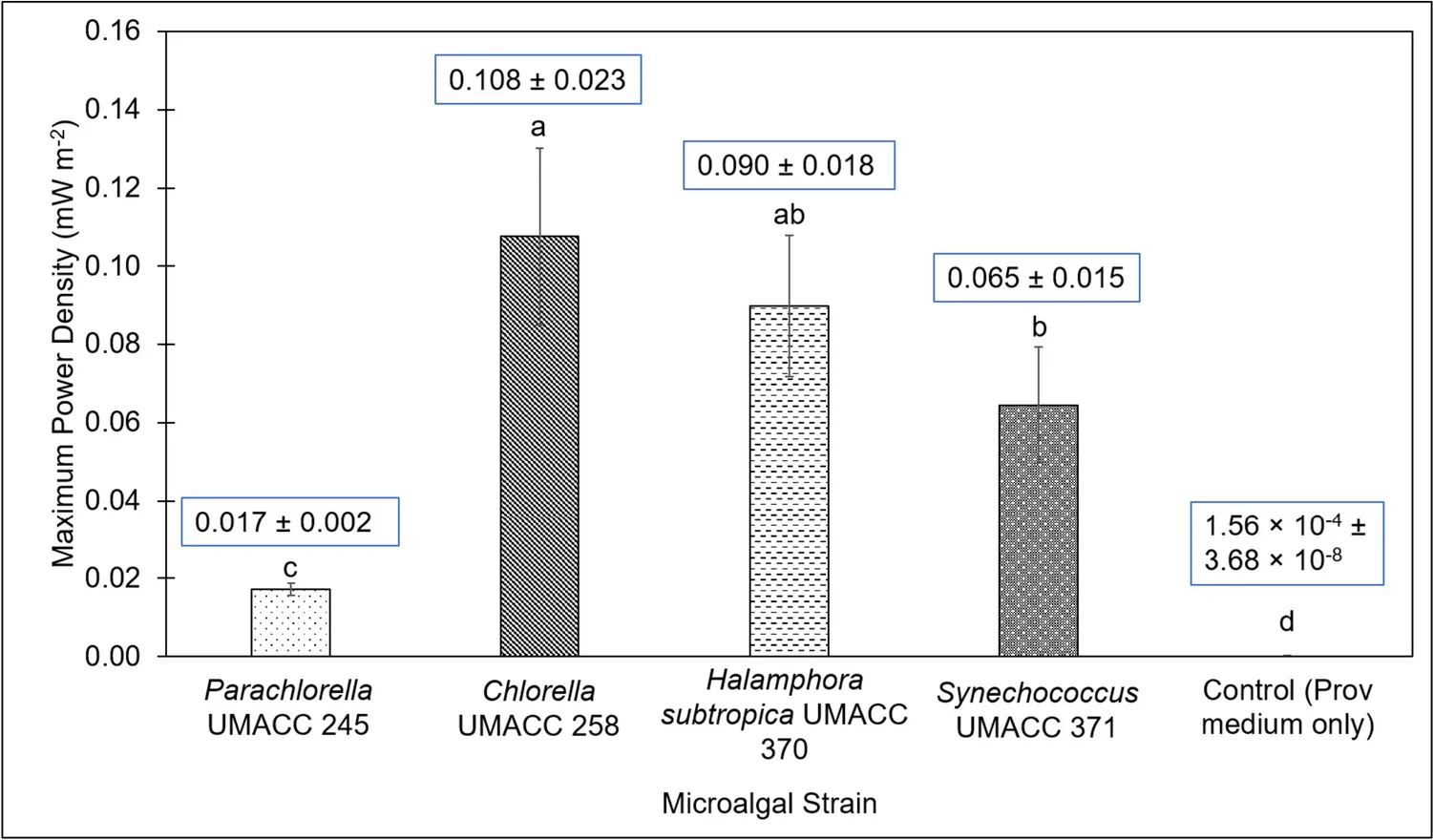
Comparison of maximum power density produced by each microalgal strain and the control (Prov medium only) in the BPV platform; data as means ± S.D (*n* = 5). Difference between alphabets indicate significant differences between different strains (ANOVA, Tukey HSD test, *p* < 0.05)

Figure 3 presents the comparison of the maximum power density produced by the four microalgal strains at an irradiance level of 90 µmol photons m^−2^ s^−1^ on Day 4.

Among all the microalgal strains, the highest power density of 0.108 ± 0.023 mW m^−2^ was produced by *Chlorella* UMACC 258, followed by *Halamphora subtropica* UMACC 370 (0.090 ± 0.018 mW m^−2^), *Synechococcus* UMACC 371 (0.065 ± 0.015 mW m^−2^) and *Parachlorella* UMACC 245 (0.017 ± 0.002 mW m^−2^). The average maximum power density generated by the Control was 1.56 × 10^−4^ ± 3.68 × 10^−8^ mW m^−2^, which was comparatively lower than any of the power densities produced by the microalgal strains. ANOVA tests revealed that there were overall significant differences in the maximum power density between the strains (ANOVA, *p* < 0.05).

On the other hand, the highest current density of 1.412 mA m^−2^ was produced by *Chlorella* UMACC 258 as well; this was followed by *Halamphora subtropica* UMACC 370 (1.164 ± 0.075 mA m^−2^), *Synechococcus* UMACC 371 (1.157 ± 0.104 mA m^−2^) and *Parachlorella* UMACC 245 (0.423 ± 0.037 mA m^−2^). Current densities obtained between the different strains were statistically significantly different from each other (ANOVA, *p* < 0.05).

### Content of photosynthetic pigments

#### Chl-a and total carotenoid contents

**Fig. 4:**
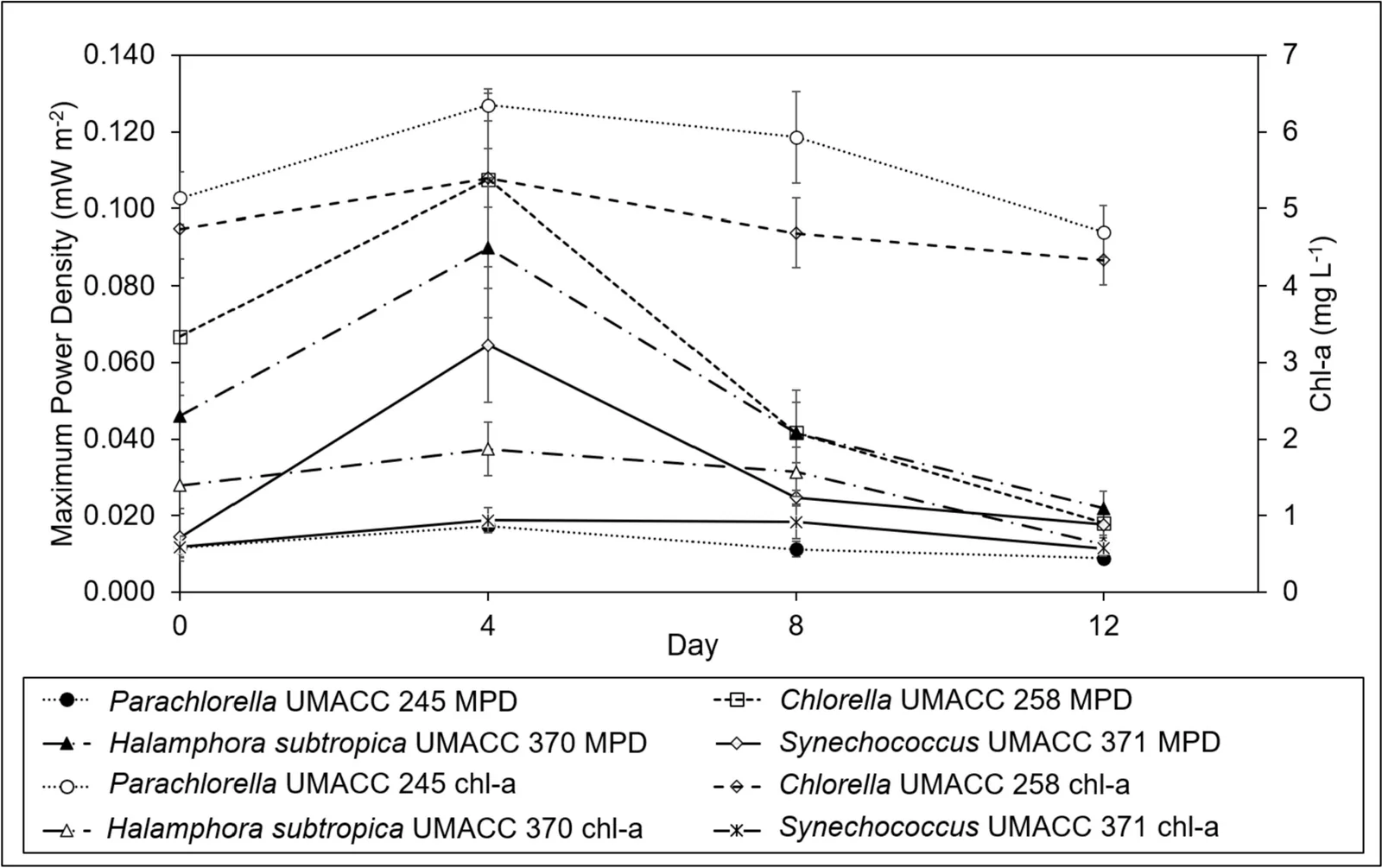
Comparison between the maximum power density (MPD) and the chl-a content of the strains on days 0, 4, 8 and 12 of the experiment; data as means ± S.D (*n* = 5)

Figure 4 shows an increase in chl-a content for all strains between Day 0 and Day 4, after which a decrease was recorded on Day 8 and subsequently, Day 12. A similar trend can also be observed for power density on respective days.

The Pearson product-moment correlation tests revealed that there was a positive relationship between the maximum power density and chl-a content for all the strains, which were statistically significant (*p* < 0.05): *Parachlorella* UMACC 245 (*r* = 0.664), *Chlorella* UMACC 258 (*r* = 0.613), *Halamphora* UMACC 370 (*r* = 0.691) and *Synechococcus* UMACC 371 (*r* = 0.450). These results imply that a higher chl-a content leads to higher power density.

Both *Parachlorella* UMACC 245 and *Chlorella* UMACC 258 recorded remarkably higher chl-a content than that of *Halamphora subtropica* UMACC 370 and *Synechococcus* UMACC 371 (Table 1). The highest chl-a contents were recorded on Day 4, with *Parachlorella* UMACC 245 (6.351 ± 0.206 mg L^−1^) producing the largest content, followed by *Chlorella* UMACC 258 (5.399 ± 0.388 mg L^−1^), *Halamphora subtropica* UMACC 370 (1.867 ± 0.349 mg L^−1^) and *Synechococcus* UMACC 371 (0.938 ± 0.164 mg L^−1^). Total carotenoid content on Day 4 was 3.032 ± 0.244 mg L^−1^ and 2.807 ± 0.149 mg L^−1^ for *Parachlorella* UMACC 245 and *Chlorella* UMACC 258, respectively. Similar to chl-a, the total carotenoid contents for *Halamphora subtropica* UMACC 370 (0.974 ± 0.229 mg L^−1^) and *Synechococcus* UMACC 371 (1.044 ± 0.062 mg L^−1^) were lower than the green microalgal strains.

**Table 1:** Chl-a and total carotenoid content of the microalgal strains on respective days of the experiment at irradiance level of 90 µmol photons m^−2^ s^−1^, data as means ± S.D (*n* = 5). Difference between alphabets indicates significant differences between different days (ANOVA, Tukey HSD test, *p* < 0.05)

Day	Chl-a content of strains (mg L^−1^)				Carotenoid content of strains (mg L^−1^)			
	*Parachlorella* UMACC 245	*Chlorella* UMACC 258	*Halamphora subtropica* UMACC 370	*Synechococcus* UMACC 371	*Parachlorella* UMACC 245	*Chlorella* UMACC 258	*Halamphora subtropica* UMACC 370	*Synechococcus* UMACC 371
0	5.137 ± 0.336^c^	3.743 ± 0.389^ cd^	1.398 ± 0.302^ef^	0.591 ± 0.140^ g^	2.665 ± 0.158^abc^	2.118 ± 0.312^d^	0.564 ± 0.136^ fg^	0.752 ± 0.091^ef^
4	6.351 ± 0.206^a^	5.399 ± 0.388^bc^	1.867 ± 0.349^e^	0.938 ± 0.164^ fg^	3.032 ± 0.244^a^	2.807 ± 0.149^ab^	0.974 ± 0.229^e^	1.044 ± 0.062^e^
8	5.933 ± 0.598^ab^	4.686 ± 0.452^ cd^	1.572 ± 0.317^ef^	0.917 ± 0.212^ fg^	2.918 ± 0.152^a^	2.242 ± 0.194^d^	0.828 ± 0.187^ef^	0.963 ± 0.086^ef^
12	4.705 ± 0.329^ cd^	4.339 ± 0.323^d^	0.628 ± 0.119^ g^	0.517 ± 0.096^ g^	2.447 ± 0.215^bcd^	2.288 ± 0.267^ cd^	0.281 ± 0.043^ g^	0.804 ± 0.056^ef^

#### PBP content

While PBPs are known as accessory pigments for photosynthesis in blue-green algae, they are still important for the photosynthetic activity of cyanobacterial cells.

Table 2 presents the PBP content of *Synechococcus* UMACC 371 obtained during the experiment. ANOVA tests revealed there to be statistically significant differences between each PBP content (PC, APC and PE) obtained on various days of the experiment (*p* < 0.05).

**Table 2:** PBP content of *Synechococcus* UMACC 371 strain on respective days of the experiment at irradiance level of 90 µmol photons m^−2^ s^−1^, data as means ± S.D (*n* = 5). Difference between alphabets indicate significant differences between different days (ANOVA, Tukey HSD test, *p* < 0.05)

	PBP pigment (µg L^−1^)		
Day	PC	APC	PE
0	3.886 ± 0.571^b^	2.850 ± 0.701^b^	3.366 ± 0.823^b^
4	5.754 ± 0.529^a^	4.952 ± 0.677^a^	5.693 ± 0.800^a^
8	3.200 ± 0.612^b^	2.580 ± 0.434^bc^	2.701 ± 0.487^b^
12	2.909 ± 0.490^b^	1.601 ± 0.356^c^	2.244 ± 0.400^b^

The highest PC (5.754 ± 0.529 µg L^−1^), APC (4.952 ± 0.677 µg L^−1^) and PE (5.693 ± 0.800 µg L^−1^) contents were found on Day 4, after which the contents of all three PBP pigments decreased with time (Day 8 and Day 12) in a similar trend to chl-a and carotenoid contents. The contents of both PC and PE were measured to be greater than APC. However, an ANOVA test reported there to be no significant difference (*p* > 0.05) between PC, APC and PE contents obtained on Day 4.

### Photosynthetic performance using PAM fluorometer measurements

#### Maximum quantum efficiency, F_v_/F_m_

According to Figure S1 and Table S2, *F*_v_/*F*_m_ values of *Synechococcus* UMACC 371 were significantly lower (*p* < 0.05) throughout the course of the experiment compared to the other strains.

The highest *F*_v_/*F*_m_ values obtained by all the strains were on Day 0 and Day 4 of the experiments. For all the strains, there is an increase in the value of *F*_v_/*F*_m_ from Day 0 to Day 4 before a decline on Day 8 and Day 12. The highest *F*_v_/*F*_m_ value recorded was by *Parachlorella* UMACC 245 (0.494 ± 0.037), closely followed by *Chlorella* UMACC 258 (0.472 ± 0.030), *Halamphora subtropica* UMACC 370 (0.379 ± 0.036) and *Synechococcus* UMACC 371 (0.284 ± 0.033) on Day 4.

#### Alpha (α)

Overall, *Chlorella* UMACC 258 experienced the highest alpha value of 0.477 ± 0.063 on Day 4, the exponential phase. According to Figure S2 and Table S3, all strains experienced a drop in alpha values as the days progressed from Days 4 to 12, suggesting the reduced health of the algal cells. *Parachlorella* UMACC 245 (0.403 ± 0.044) produced the second-highest alpha value on Day 4, followed by *Halamphora subtropica* UMACC 370 (0.354 ± 0.019) and *Synechococcus* UMACC 371 (0.316 ± 0.024). Although the maximum power density of *Synechococcus* UMACC 371 (0.065 ± 0.015 mW m^−2^) was 73.85% higher than that of *Parachlorella* UMACC 245 (0.017 ± 0.002 mW m^−2^), its alpha value was 21.59% lower.

#### Maximum relative electron transport rate, rETR_max_

On Day 4 when power outputs for all the strains were highest, the rETR_max_ values for all strains were above 150 µmol electrons m^−2^ s^−1^ with the exception of *Halamphora subtropica* UMACC 370 (Figure S3 and Table S4). The highest rETR_max_ values were obtained by *Chlorella* UMACC 258 (167.449 ± 21.068 µmol electrons m^−2^ s^−1^) and *Parachlorella* UMACC 245 (165.683 ± 29.496 µmol electrons m^−2^ s^−1^), followed by *Synechococcus* UMACC 371 (150.411 ± 23.891 µmol electrons m^−2^ s^−1^) and *Halamphora subtropica* UMACC 370 (53.126 ± 3.532 µmol electrons m^−2^ s^−1^). Values of rETR_max_ for *Parachlorella* UMACC 245 and *Chlorella* UMACC 258 on Day 4 were similar and had no significant difference (ANOVA, *p* > 0.05).

#### Photo-adaptive index, E_k_

According to Figure S4 and Table S5, all strains experienced highest *E*_k_ values on Day 4: *Parachlorella* UMACC 245 (410.219 ± 32.031 µmol photons m^−2^ s^−1^), *Chlorella* UMACC 258 (358.240 ± 84.023 µmol photons m^−2^ s^−1^), *Halamphora subtropica* UMACC 370 (176.148 ± 18.410 µmol photons m^−2^ s^−1^) and *Synechococcus* UMACC 371 (483.111 ± 113.587 µmol photons m^−2^ s^−1^). By Day 12, there was a reduction in *E*_k_ values by 28.98%, 33.04%, 9.02% and 26.41% for *Parachlorella* UMACC 245, *Chlorella* UMACC 258, *Halamphora subtropica* UMACC 370 and *Synechococcus* UMACC 371, respectively.

#### Non-photochemical quenching, NPQ

NPQ was active in all the strains except for *Synechococcus* UMACC 371 (Figure S5). The highest NPQ values exerted by the four strains during exponential phase (Day 4) were 1.883 ± 0.338 (*Halamphora subtropica* UMACC 370), 0.861 ± 0.158 (*Chlorella* UMACC 258), 0.660 ± 0.040 (*Parachlorella* UMACC 245) and 0.236 ± 0.0275 (*Synechococcus* UMACC 371).

### Electrochemical studies

**Fig. 5:**
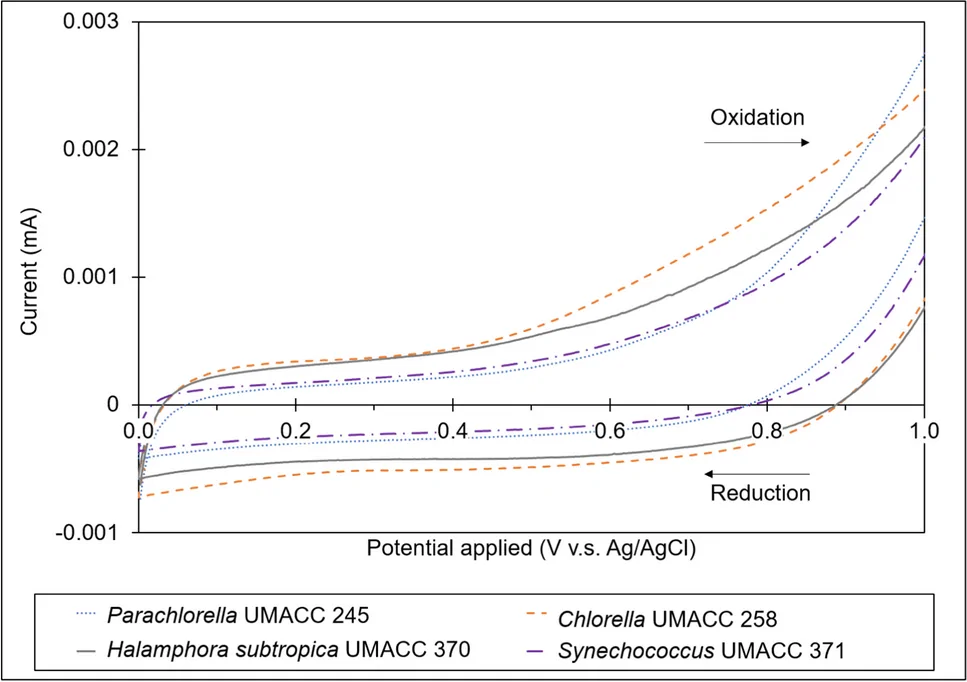
Comparison of cyclic voltammograms for the four different algal strains with a potential window from 0 to + 1 V and a scan rate of 5 mVs.^−1^

Figure 5 shows cyclic voltammograms for the respective algal strains as a result of conducting CV. Between potentials 0.1 and 0.9 V during the forward scan, *Chlorella* UMACC 258 biofilm-loaded on carbon paper anode was able to generate the highest current among the four strains. This was followed by *Halamphora subtropica* UMACC 370, *Synechococcus* UMACC 371 and *Parachlorella* UMACC 245. This trend observed is consistent with that of current density values reported in Table S1.

### Cell growth on anode surface using FESEM

**Fig. 6:**
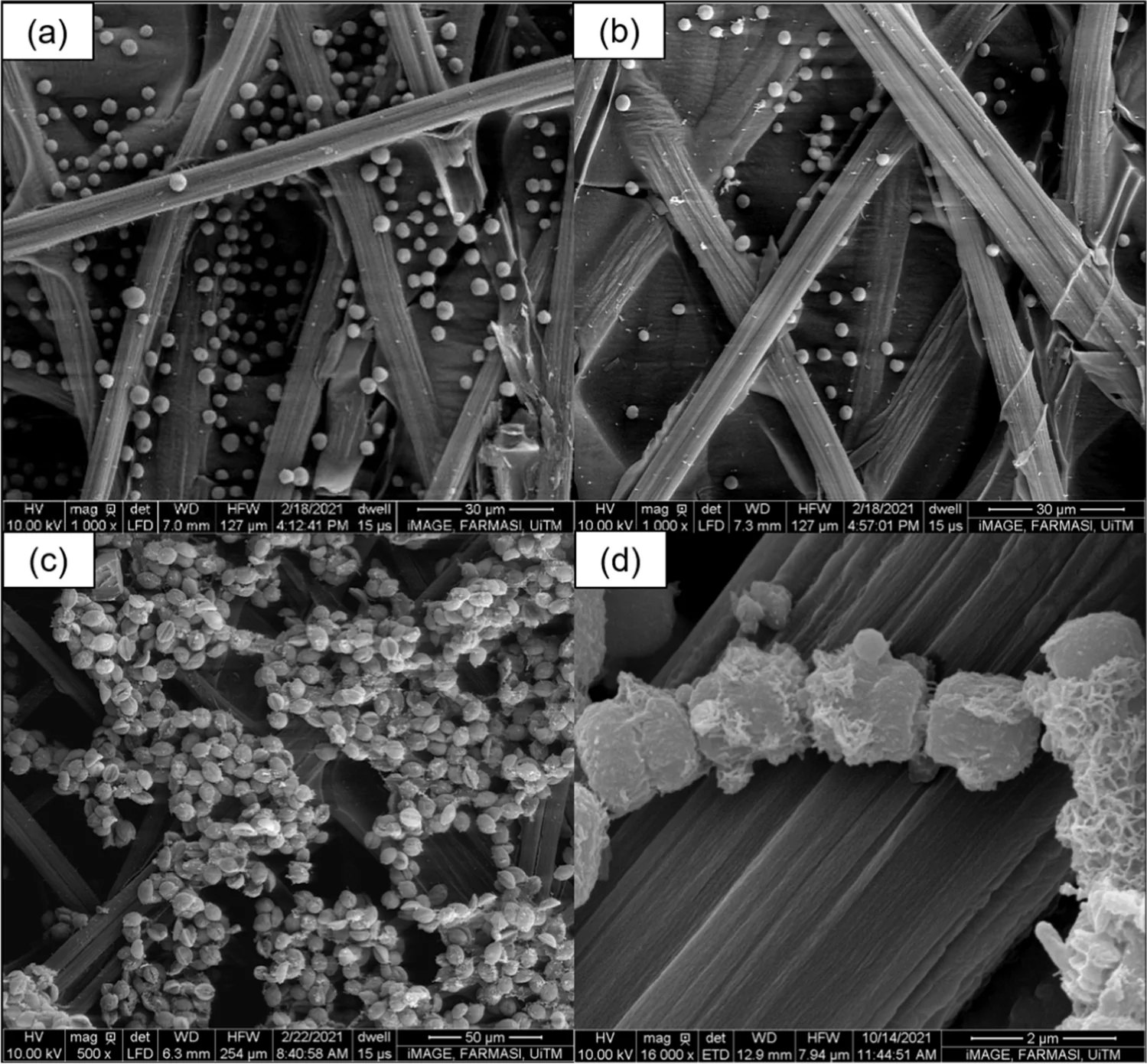
FESEM images of **a** *Parachlorella* UMACC 245 cells lodged within the fibre structures of the carbon paper anode, scale bar = 30 µm; **b** *Chlorella* UMACC 258 growing on carbon anode surface, scale bar = 30 µm; **c** Hala*mphora subtropica* UMACC 370 cells growing on fibre structure of anode, scale bar = 50 µm and **d** biofilm formation of *Synechococcus* UMACC 371 cells, scale bar = 2 µm

As seen in Fig. 6, the algal cells grew favourably on the surface as well as within the intricate fibre structures of the carbon paper anode. Figure 6a and b, in particular, shows that the cells were able to move deep into the grooves of the carbon anode and grow in abundance. Figure 6d shows the biofilm formation of *Synechococcus* UMACC 371 cells, especially on those situated on the right side of the image.

## Discussion

### Comparison of power output generated by algal strains and control

The microalgal strains arranged in descending order according to maximum power density are *Chlorella* UMACC 258 (0.108 ± 0.023 mW m^−2^), *Halamphora subtropica* UMACC 370 (0.090 ± 0.018 mW m^−2^), *Synechococcus* UMACC 371 (0.065 ± 0.015 mW m^−2^) and *Parachlorella* UMACC 245 (0.017 ± 0.002 mW m^−2^). Although there are other studies that reported greater generation of power output from BPV platforms, it is very likely that the difference is attributable to the microalgal species used, cell density, setup process of the BPV platforms and the BPV materials used such as the anode and PEM. The difference in values of power output produced by the four microalgal strains used in this study can be explained by their photosynthetic pigment contents as well as photosynthetic efficiency of cells, which will be discussed in the following sub-sections.

The maximum power density generated by the Control was 1.56 × 10^−4^ ± 3.68 × 10^−8^ mW m^−2^ (Table S1). This value is significantly smaller (*p* < 0.05) than any of the maximum power densities generated by the microalgal strains. Even though Prov medium is saline (30 ppt), and it contains sodium (Na^+^) and chloride (Cl^−^) electrolytes that made electrical conductivity possible, the low current and power densities produced by the Control imply that the Prov medium has a negligible contribution to the power output obtained in the experiments.

### Pigment content

#### Correlation between Chl-a content and power output

Microalgae, in general, experience four phases of growth: lag phase, log or exponential phase, stationary phase and death phase. Based on many previous studies by the Universiti Malaya Algae Research Lab, microalgal cells undergo a life cycle of approximately 12 days, so the 4-day intervals are to take the relevant measurements during which the cells undergo different phases. (Ng et al. 2014a, 2014b, 2017, 2018; Thong et al. 2019, 2023). As presented in Fig. 4, there was a general increasing trend for both power density and chl-a content of each strain between Day 0 and Day 4, after which both measurements decreased on Day 8 and Day 12. The resulting increase in the chl-a content on Day 4 indicates a higher concentration of cells and algal biomass compared to Day 0, possibly due to the sufficient supply of nutrients and their acclimation to the environment within the BPV platforms. This also corresponds with an increase in power output. By Day 8 and Day 12, chl-a content had decreased which signified the likely death of some algal cells due to nutrient depletion.

On Day 4, *Parachlorella* UMACC 245 (6.351 ± 0.206 mg L^−1^) produced the highest chl-a content out of the four strains, followed by *Chlorella* UMACC 258 (5.399 ± 0.388 mg L^−1^). Both *Halamphora subtropica* UMACC 370 (1.867 ± 0.349 mg L^−1^) and *Synechococcus* UMACC 371 (0.938 ± 0.164 mg L^−1^) produced considerably lower contents. Being green algae, *Parachlorella* UMACC 245 and *Chlorella* UMACC 258 only rely on chl-a and accessory pigments namely chl-b and carotenoids to capture light for photosynthesis.

Chl-a absorbs light in the red and blue regions of the electromagnetic spectrum and reflects green colour, therefore giving the algae a green appearance (Hosikian et al. 2010). Therefore, it can be presumed that both *Parachlorella* UMACC 245 and *Chlorella* UMACC 258 would have more abundant chl-a compared to *Halamphora subtropica* UMACC 370 and *Synechococcus* UMACC 371, which are phenotypically brown and blue-green in colour, respectively. *Halamphora subtropica* UMACC 370 and *Synechococcus* UMACC 371 consist of other photosynthetic pigments to assist with light capture. *Halamphora subtropica* UMACC 370 comprises of chl-c and fucoxanthin in addition to chl-a, all of which combine to form chlorophyll-fucoxanthin protein (FCP) complexes that play the role of harvesting light instead of LHCs typically found in green algae (Gelzinis et al. 2015). On the other hand, *Synechococcus* UMACC 371 contains accessory pigments, PBPs, other than chl-a.

Despite the fact that chl-a content of *Parachlorella* UMACC 245 (6.351 ± 0.206 mg L^−1^) was higher than that of *Chlorella* UMACC 258 (5.399 ± 0.388 mg L^−1^), the power output obtained by the former was slightly lower than the latter. The reason may be that the concentration of chl-a pigments may have not been the only factor that affected the power output producible because the photosynthetic efficiency of cells could have been another factor that influenced the power output. Photosynthetic efficiency of the strains is compared and discussed in the “Photosynthetic performance of strains” section.

#### PBP pigments in *Synechococcus* UMACC 371

In conditions where light is sufficient, PBP pigments help to absorb light within the 500–600 nm range and transferred with high efficiency of 80–90% to chlorophyll in order to provide supplementary energy to the photosynthetic centres of the cells. On the other hand, in low-light environments, the PBP pigments help to maintain the photosynthesis rate of the cyanobacterial cells (Śliwińska-Wilczewska et al. 2020), allowing the microalgae to survive in environments where light is scarce.

According to Table 2, PC and PE contents were measured to be greater than APC. This implies that *Synechococcus* UMACC 371 may be PC- and PE-rich, though ANOVA tests had revealed no significant differences between PC, APC and PE contents on Day 4 (*p* > 0.05).

Nevertheless, the determination of the concentration of PBP verifies the presence of PBPs in *Synechococcus* UMACC 371 and their role in light harvest alongside chl-a and carotenoid pigments.

### Photosynthetic performance of strains

#### Cell stress indicated by maximum quantum efficiency, F_v_/F_m_

*F*_v_/*F*_m_ measures the concentration of PSII reaction centres that are open to absorb light (Genty et al. 1989). It is indicative of any physiological stress experienced by the microalgal cells (Maxwell and Johnson 2000). Various studies have reported *F*_v_/*F*_m_ values ranging from 0.10 to 0.65 for natural populations of microalgae (Reeves et al. 2011). Results from the current study showed that the strains were the healthiest on Day 4 when acclimation had occurred in the BPV platforms. The overpopulation of cells and nutrient depletion may have caused a drop in the *F*_v_/*F*_m_ values on Day 8, and a further drop on Day 12.

*Synechococcus* UMACC 371 had considerably lower *F*_v_/*F*_m_ values throughout the course of the experiment compared to the other strains, ranging between 0.116 and 0.284. This is, however, typical for blue-green algae when the PAM fluorometer measurement approach is used as low *F*_m_ and high *F*_0_ values are obtained. The low *F*_v_/*F*_m_ values of cyanobacteria are mainly caused by interfering background fluorescence emitted by the phycobilisomes or free PBPs (Papageorgiou et al. 2007; Schuurmans et al. 2015). Phycobilisomes, made of stacks of PBPs and linker polypeptides, are light-harvesting complexes (LHCs) of cyanobacteria attached to the thylakoid membranes of the cyanobacteria. In a study by Campbell et al. (1998) involving a wild-type *Synechococcus* sp. PCC 7942 strain and a mutated PCC 7942 strain lacking PC, a positive correlation between *F*_0_ and PC content was found i.e., a lower *F*_0_ observed in the mutated strain that lacked PC. Because the fluorescence emission by PBPs contributes to the value of *F*_0_ but not *F*_v_ = *F*_m_ – *F*_v_, then a change in the PBP concentration in the cell is presumed to affect the *F*_v_/*F*_m_ value. Moreover, the recorded fluorescence of *Synechococcus* UMACC 371 may have also originated from the chl-a of PSI, suggesting that the PAM signal obtained may have been contributed by a combination of chl-a of PSI and PSII and phycobilisomes (Campbell et al. 1998; Kirilovsky 2015).

#### Alpha (α), maximum relative electron transport rate, rETR_max_, and photo-adaptive capacity of strains

Alpha (α) measures the light-harvesting efficiency of the microalgae; the rETR_max_ is an indication of the maximum capacity for photosynthesis (Ng et al. 2017). Overall, *Chlorella* UMACC 258 experienced the highest alpha (0.477 ± 0.063) and rETR_max_ (167.449 ± 21.068 µmol electrons m^−2^ s^−1^) on Day 4. This finding suggests that it has the highest efficiency in light absorption for the excitation of electrons, and the least amount of delay in their rates of photosynthetic electron transport through PSII (Malapascua et al. 2014) compared to the other three strains. This can be corroborated by producing the highest power output of 0.108 ± 0.023 mW m^−2^ (Fig. 3).

*E*_k_ represents the saturation irradiance (Malapascua et al. 2014) and indicates the ability of the algal cells or biofilms to adapt to high light intensities (Ng et al. 2014b). The high values of *Parachlorella* UMACC 245, *Chlorella* UMACC 258 and *Synechococcus* UMACC 371 infer these strains’ favourable adaptative capacity to high light irradiances. While the values of *Halamphora subtropica* UMACC 370 were comparatively lower than the other strains, this strain seemed to have the most stable *E*_k_ as its values fluctuated between 141.614 and 176.148 µmol photons m^−2^ s^−1^ throughout the course of the experiment (Table S5). This may be due to the carotenoids diadinoxanthin (Dd) and diatoxanthin (Dt), which form the total xanthophyll pool for diatoms and work as their photoprotective system. The pool size of Dd plus Dt has been discovered to remain constant on timescales of seconds to hours and increase on timescales of hours to days in response to high irradiances (Moline 1998).

#### Photoprotection during intense irradiances

PSII is the most light-sensitive complex in the photosynthetic apparatus of microalgae (Vass and Aro 2007; Tyystjärvi 2008). Photodamage brought upon by high-intensity solar radiation negatively impacts the proteins of PSII, thereby resulting in lower CO_2_ fixation, as well as poorer growth and development of the algal cells (Pathak et al. 2019). In microalgae, NPQ helps to protect them from photodamage by dissipating excess energy as heat as a form of photoprotection (Ng et al. 2014b). It is associated with the xanthophyll cycle (XC) in which the de-epoxidation of xanthophylls occurs (Lavaud 2007).

Unlike the other three strains, *Synechococcus* UMACC 371 showed very low NPQ values regardless of the PAR condition exposed. These low values imply the microalga’s weak capacity for photoprotection, but its recorded carotenoid content (Table 1) and very high *E*_k_ values (Table S5) propose that it may in fact have tolerance to high light irradiances. This phenomenon may have been due to its “orange carotenoid protein (OCP)”-dependent NPQ mechanism that involves the light-activation of the OCP found in phycobilisomes (Boulay et al. 2008), which is unlike the other algal strains. The OCP is orange under resting conditions. Upon light absorption by the OCP, structural changes are made in the carotenoid and protein, which triggers the conversion of the dark stable orange form into a red relatively unstable form. The red form would then accumulate in situations where cyanobacterial cells are exposed to intense light in order to induce photoprotection (Wilson et al. 2008).

In the case of diatom *Halamphora subtropcia* UMACC 370, it exerted the strongest NPQ among all the strains, with its peak NPQ value almost reaching 2.0. This finding is in agreement with a number of studies which have reported on diatoms’ ability to generally exert a strong NPQ (Lavaud et al. 2002; Ruban et al. 2004; Lavaud 2007), giving them the ability to respond to quick changes in light intensity or irradiance. This is mediated by (1) light-harvesting complex stress-related (LHCSR) proteins termed “light-harvesting complex protein binding fucoxanthin/light-harvesting complex protein x (Lhcf/Lhcx proteins)”; and (2) rapid, extensive de-epoxidation from Dd to Dt (Lavaud and Goss 2014).

In green algae, the NPQ activity is a result of the chl-a fluorescence mechanism. *Parachorella* UMACC 245 was able to exhibit NPQ in a steadily increasing order with a possible plateau after a PAR condition of 800 µmol photon m^−2^ s^−1^, indicative of a likely NPQ saturation. In comparison, the NPQ in *Chlorella* UMACC 258 remained roughly constant between PAR conditions of 150 and 250 µmol photon m^−2^ s^−1^ before steadily rising. Then, there was a sharp increase in NPQ activity at approximately 700 µmol photon m^−2^ s^−1^. The slow increase in NPQ activity observed in *Chlorella* UMACC 258 during lower irradiances could be explained by its initial reliance on state transitions as a form of photoprotective mechanism before the XC was triggered in response to more intense light (Garcia-Mendoza et al. 2002). The de-epoxidation of carotenoids violaxanthin (Vx) to zeaxanthin (Zx) via an intermediate antheraxanthin (Ax) occurred (Quaas et al. 2015).

### Electrochemical behaviour of cells

All strains were able to generate oxidation curves (Fig. 5) which suggests the suitability of their biofilms to provide direct electron donation to the carbon paper anode. The electrochemical behaviour of the biofilm-loaded carbon paper anodes is likely to be due to the prevalence of the electrochemically active proteins on the cellular surface of the microalgae (Wu et al. 2013; Karthikeyan et al. 2019).

*Chlorella* UMACC 258 produced the highest current among the four strains. This occurrence may be due to high biocompatibility between the *Chlorella* UMACC 258 strain and the carbon paper anode and can be supported by the strain’s high rETR_max_ value (167.449 ± 21.068 µmol electrons m^−2^ s^−1^) obtained on Day 4 when it was the healthiest. Furthermore, this suggests the presence of electrochemically active proteins on the cellular surface (Karthikeyan et al. 2019). As the biofilms of the other three algal strains also generated currents, it can be said that the algal cells are also biocompatible with the carbon paper anodes, despite not being as substantial as that with *Chlorella* UMACC 258.

### Biocompatibility of algal cells on carbon paper anodes

Carbon paper was selected as the material for the anode due to several properties. First is due to their biocompatibility. Second, it is able to offer a high specific surface area for growth of algal biofilms. Third, it costs comparatively less than other known anode materials such as ITO (tin indium oxide) and rGO (reduced graphene oxide). The FESEM images demonstrate information on the growth of the biofilms on the carbon paper anodes. The growth of biofilms on the anodes indicates their biocompatibility with the anodes. This close relationship between the two then results in the generation of power output and shows the ability of the biofilms to produce power output in BPV platforms without the use of exogenous mediators, which is in agreement with the study by Mccormick et al. (2011). Moreover, they show the presence of biofilms that are most likely responsible for the electrochemical behaviours observed in the electrochemical studies of the abovementioned sub-section.

In summary, the maximum power density generated in this study was 0.108 ± 0.023 mW m^−2^ by *Chlorella* UMACC 258 on Day 4 of the experiment. This is followed by the *Halamphora subtropica* UMACC 370 (0.090 ± 0.018 mW m^−2^), *Synechococcus* UMACC 371 (0.065 ± 0.002 mW m^−2^) and *Parachlorella* UMACC 245 (0.017 ± 0.015 mW m^−2^). The Pearson product-moment correlation test reported a positive relationship between the power density and the chl-a meaning that a greater algal biomass would lead to a greater power density (*p* < 0.05). *Chlorella* UMACC 258’s high power output was attributed to high photosynthetic performance in terms of *F*_v_/*F*_m_, alpha, rETR_max_ and *E*_k_.

The results from this study show the ability of all the algal strains tested to produce bioelectricity by using BPV platforms. These findings present additional knowledge to the types of algal strains that could be used in BPV platforms and could be useful for the future optimisation of the BPV platform. Marine microalgae may offer certain advantages over freshwater microalgae. Because they live in saltwater environments, future applications allow for marine microalgae-powered BPV platforms to be installed in the sea and would not take up arable land areas. They may also be useful in monitoring water quality and other environmental factors in seaweed, fish and shrimp farms. A few potential modifications could be made to the BPV system to enhance power output. One would be to increase the surface area of the anode in order to facilitate more biofilm growth. Other modifications include increasing the overall size of the BPV system to accommodate more culture volume, and attaching a supercapacitor to a BPV system so that electrons discharged during the water-splitting reaction could be stored and accumulated.

## Supplementary Information

Below is the link to the electronic supplementary material.

Supplementary file1 (PDF 405 KB)
