# Supplementary material for: Evaluation of selected tropical marine microalgal cultures for use in biophotovoltaic platforms
Source: Appl Microbiol Biotechnol. 2024 Jan 9;108:71. doi: 10.1007/s00253-023-12951-0 (PMC10776707; doi:10.1007/s00253-023-12951-0)
Supplement: Supplementary file 1 [file 253_2023_12951_MOESM1_ESM.pdf]

Supplementary data

Evaluation of selected tropical marine microalgal cultures for use in biophotovoltaic

Zoe Hui-Yee Tay[a], Fong-Lee Ng[b,c*], Cheng-Han Thong[b,d] , Choon-Weng Lee[a,b], G. Gnana kumar[e,f], Abdullah G. Al-Sehemi[g], Siew-Moi Phang[b,h*]

[a]Institute of Biological Sciences, Faculty of Science, Universiti Malaya, Kuala Lumpur, Malaysia

[b]Institute of Ocean and Earth Sciences (IOES), Universiti Malaya, Kuala Lumpur, Malaysia

[c]School of Biosciences, Taylor's University, Lakeside Campus, 47500, Subang Jaya, Selangor Darul Ehsan, Malaysia

[d]Institute for Advanced Studies (IAS), Universiti Malaya, Kuala Lumpur, Malaysia

[e]Department of Physical Chemistry, School of Chemistry, Madurai Kamaraj University, Madurai-625021, Tamil Nadu, India

[f]Faculty of Engineering Technology & Built Environment, UCSI University, Kuala Lumpur 56000, Malaysia

[g]Research Center for Advanced Materials Science (RCAMS), King Khalid University, Abha, 61413, Saudi Arabia

[h]Faculty of Applied Sciences, UCSI University, Kuala Lumpur, Malaysia

* Corresponding authors

E-mail: phang@um.edu.my; +603-79674610

& fonglee_ng@yahoo.com; +603-79674610

Figure S1 Fv/Fm values of all four strains on Days 0, 4, 8 and 12 of the experiment; data as means ± S.D. (n = 5)

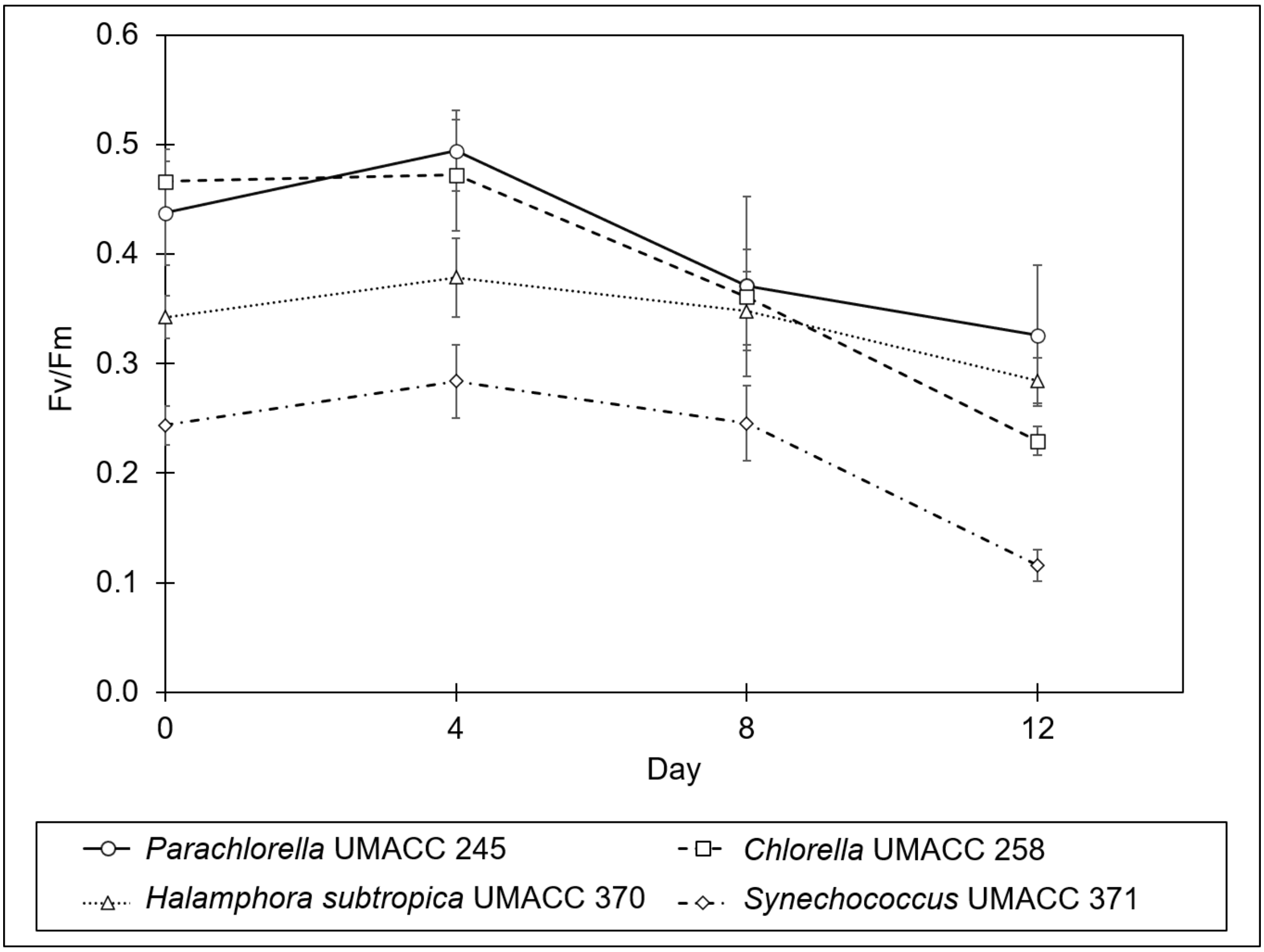

Figure S2 Comparison of the alpha values between the microalgal strains on Days 0, 4, 8 and 12 of the experiment; data as means ± S.D. (n = 5)

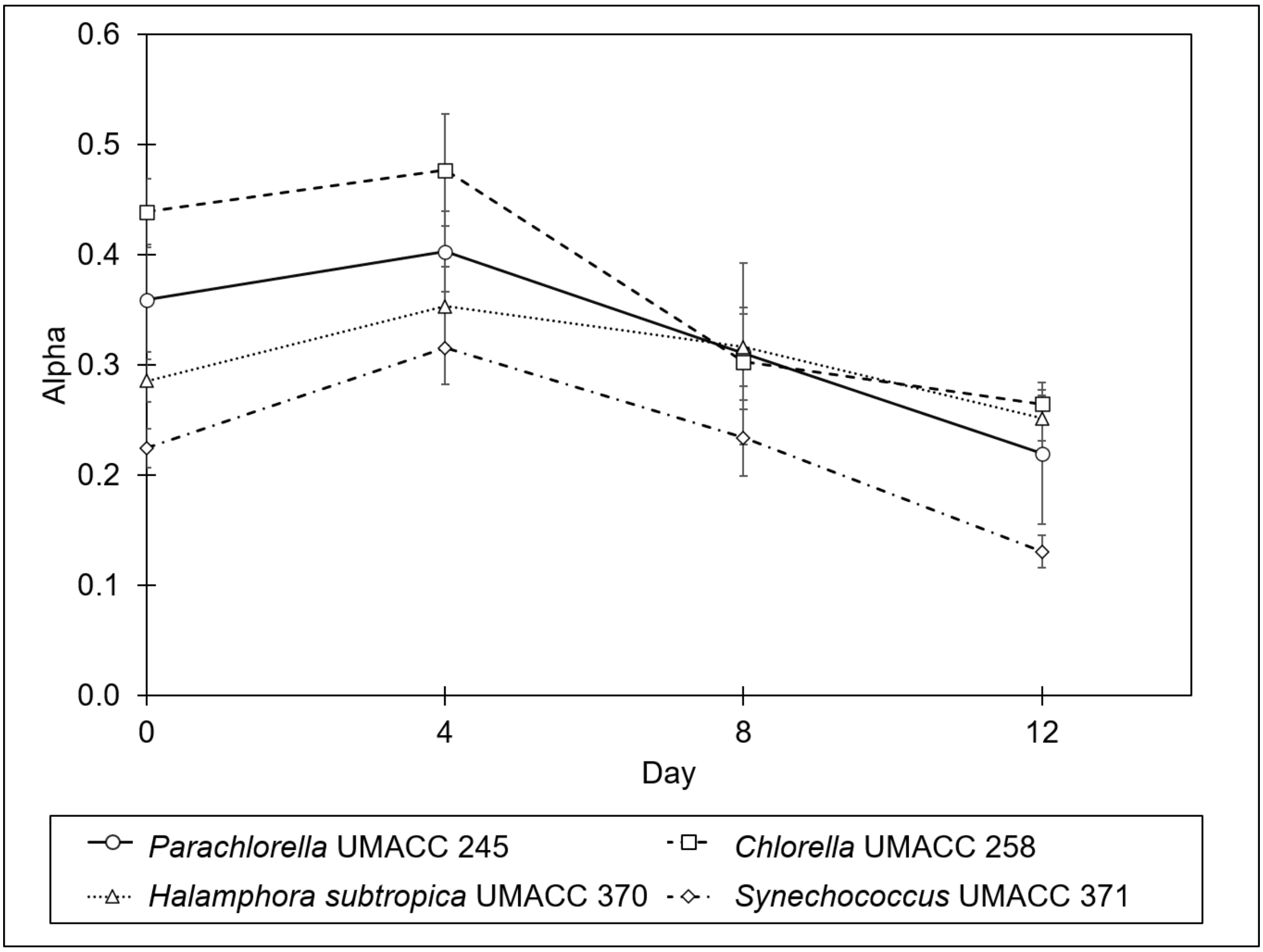

Figure S3 rETR$_{max}$ values for all strains on various days of the experiment; data as means ± S.D. (n = 5)

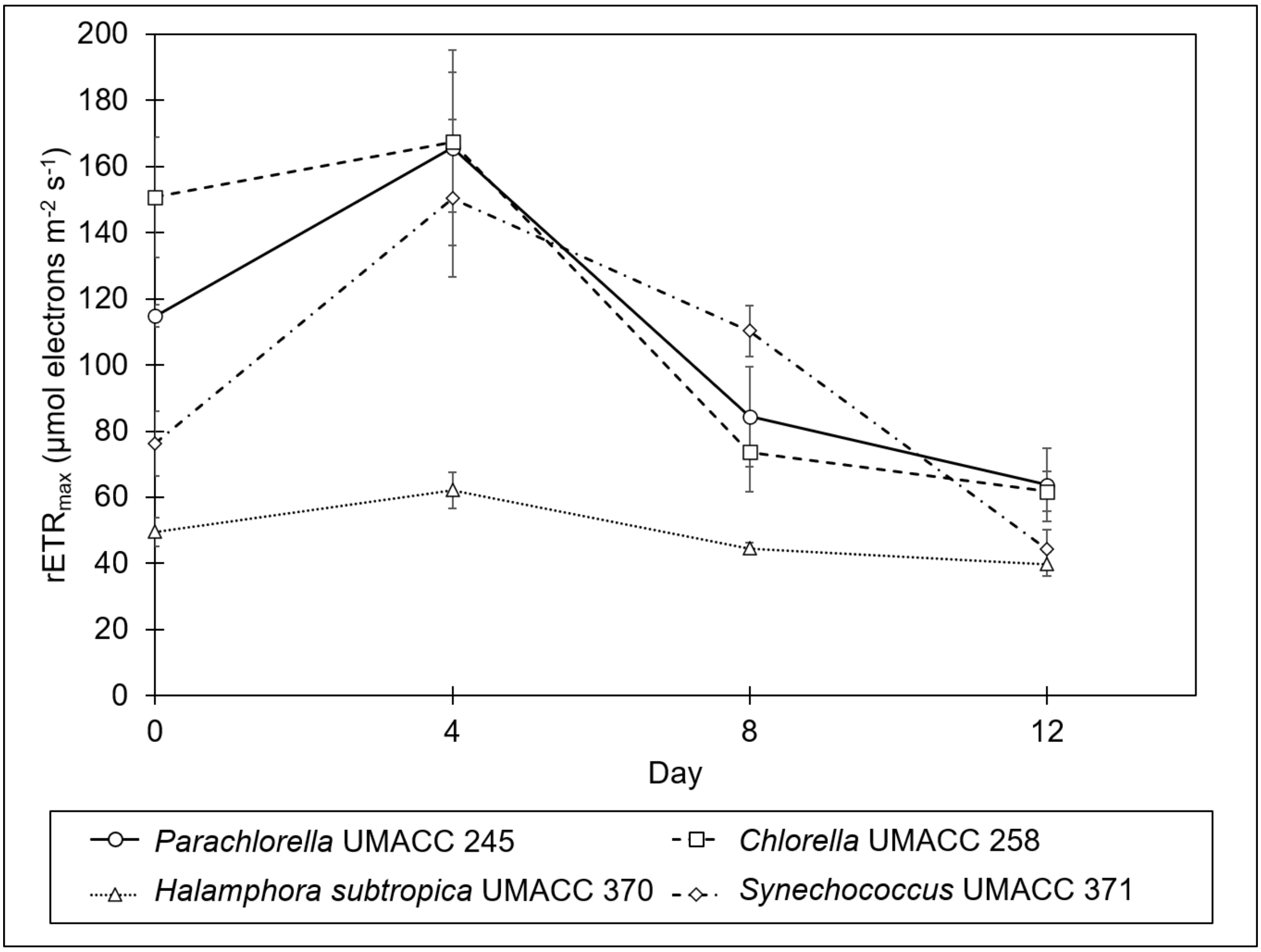

Figure S4 Comparison of the Ek values between the microalgal strains on different days of the experiment; data as means ± S.D. (n = 5)

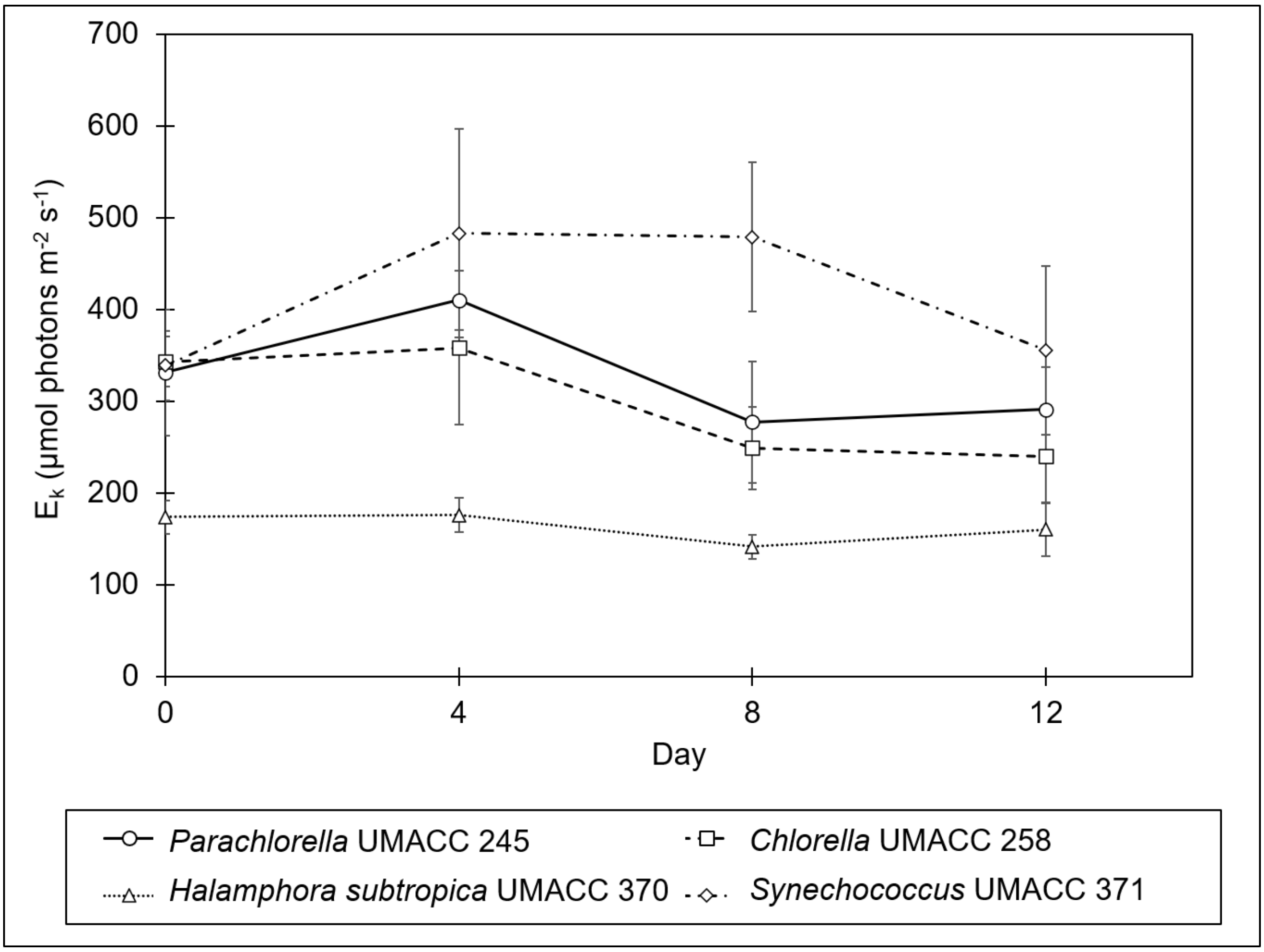

Figure S5 Comparison of the NPQ values recorded by strains on Day 4 of experiment; data as means ± S.D. (n = 5)

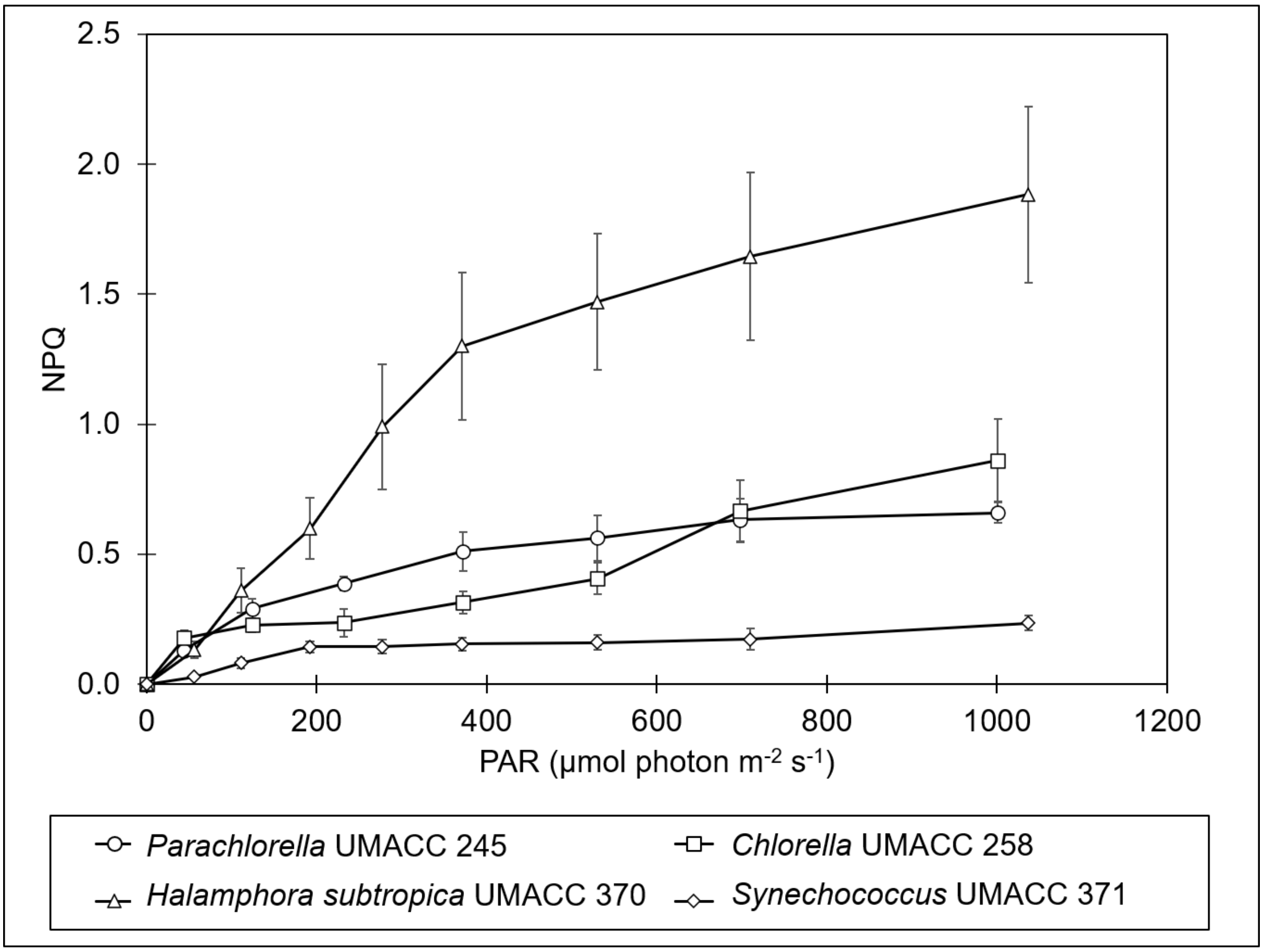

Table S1 Highest values of maximum current density and maximum power density for all strains at irradiance level of 90 µmol photons m^{-2} s^{-1}, data as means ± S.D. (n = 5). Difference between alphabets indicate significant differences between different strains (ANOVA, Tukey HSD test, $p < 0.05$)

Strain	Maximum Current Density ($mA\ m^{-2}$)	Maximum Power Density ($mW\ m^{-2}$)
Parachlorella UMACC 245	0.423 ± 0.037^{c}	0.017 ± 0.002^{c}
Chlorella UMACC 258	1.412 ± 0.124^{a}	0.108 ± 0.023^{a}
Halamphora subtropica UMACC 370	1.164 ± 0.075^{b}	0.090 ± 0.018^{ab}
Synechococcus UMACC 371	1.157 ± 0.104^{b}	0.065 ± 0.015^{b}
Control (Prov medium only)	$1.36 \times 10^{-2} \pm 2.99 \times 10^{-3\ d}$	$1.56 \times 10^{-4} \pm 3.68 \times 10^{-8\ d}$

Table S2 F_v/F_m values for all strains on respective days of the experiment, data as means ± S.D. (n = 5). Difference between alphabets indicate significant differences between different strains (ANOVA, Tukey HSD test, $p < 0.05$)

Day	F_v/F_m values for strain			
	Parachlorella UMACC 245	*Chlorella* UMACC 258	*Halamphora subtropica* UMACC 370	*Synechococcus* UMACC 371
0	0.437 ± 0.047^{abc}	0.466 ± 0.030^{ab}	0.342 ± 0.019^{de}	0.244 ± 0.018^{fg}
4	0.494 ± 0.037^{a}	0.472 ± 0.051^{a}	0.379 ± 0.036^{bcd}	0.284 ± 0.033^{efg}
8	0.370 ± 0.082^{cde}	0.361 ± 0.043^{cde}	0.348 ± 0.036^{cde}	0.245 ± 0.034^{fg}
12	0.326 ± 0.064^{def}	0.229 ± 0.013^{g}	0.284 ± 0.020^{efg}	0.116 ± 0.014^{h}

Table S3 Alpha values for all strains on respective days of the experiment, data as means ± S.D. (n = 5). Difference between alphabets indicate significant differences between different strains (ANOVA, Tukey HSD test, $p < 0.05$)

**Day**	**Alpha values for strain**			
	Parachlorella UMACC 245	*Chlorella* UMACC 258	*Halamphora subtropica* UMACC 370	*Synechococcus* UMACC 371
0	0.359 ± 0.080^{bcd}	0.439 ± 0.033^{bc}	0.286 ± 0.016^{de}	0.225 ± 0.012^{ef}
4	0.403 ± 0.044^{abc}	0.477 ± 0.063^{a}	0.354 ± 0.019^{bcd}	0.316 ± 0.024^{cde}
8	0.311 ± 0.049^{cde}	0.303 ± 0.071^{de}	0.317 ±0.031^{cde}	0.234 ± 0.030^{e}
12	0.220 ± 0.032^{ef}	0.265 ± 0.050^{de}	0.252 ± 0.028^{e}	0.131 ± 0.039^{f}

Table S4 $rETR_{max}$ for all strains on respective days of the experiment, data as means ± S.D. (n = 5). Difference between alphabets indicate significant differences between different strains (ANOVA, Tukey HSD test, $p < 0.05$)

Day	$rETR_{max}$ values for strain (μmol electrons m^{-2} s^{-1})			
	Parachlorella UMACC 245	*Chlorella* UMACC 258	*Halamphora subtropica* UMACC 370	*Synechococcus* UMACC 371
0	114.821 ± 3.389^{b}	150.773 ± 18.289^{a}	49.549 ± 4.409^{def}	76.283 ± 9.819^{cd}
4	165.683 ± 29.496^{a}	167.449 ± 21.068^{a}	62.139 ± 5.476^{cdef}	150.411 ± 23.891^{a}
8	84.393 ± 15.199^{bc}	73.609 ± 11.853^{def}	44.532 ± 1.659^{ef}	110.263 ± 7.794^{b}
12	63.690 ± 11.106^{cdef}	61.741 ± 6.096^{cdef}	39.749 ± 3.682^{f}	44.165 ± 5.895^{ef}

Table S5 E_k for all strains on respective days of the experiment; data as means ± S.D. (n = 5). Difference between alphabets indicate significant differences between different strains (ANOVA, Tukey HSD test, $p < 0.05$)

Day	E_k for strain (μmol photons m^{-2} s^{-1})			
	Parachlorella UMACC 245	*Chlorella* UMACC 258	*Halamphora subtropica* UMACC 370	*Synechococcus* UMACC 371
0	331.650 ± 68.741^{bc}	342.927 ± 27.256^{bc}	173.740 ± 18.564^{de}	338.957 ± 37.817^{bc}
4	410.219 ± 32.031^{ab}	358.240 ± 84.023^{abc}	176.148 ± 18.410^{de}	483.111 ± 113.587^{a}
8	277.330 ± 66.238^{bcd}	249.161 ± 44.840^{cde}	141.614 ± 12.884^{e}	478.755 ± 81.303^{a}
12	291.356 ± 46.063^{bcd}	239.881 ± 49.794^{cde}	160.254 ± 28.993^{de}	355.525 ± 91.491^{abc}
